# The Book World of Medicine and Science

**Published:** 1906-06-16

**Authors:** 


### The Book World of Medicine and Science.

The Cause and Prevention of Dental Caries. By J. Sim Wallace, M.D., L.D.S. (London: Baillière, Tindall, and Cox. 1906. Pp. 81. Price 2s. 6d. net.)

Though somewhat crude in style, this book contains much that is worthy of serious consideration. There is an unfortunate tendency amongst the laity—and also, it is to be feared, even among medical men, who ought to know better—to regard decay of the teeth as one of the inevitable minor ills to which mankind must submit with the best cheer available. This pernicious fatalism is the dragon which Dr. Sim Wallace sets himself out to slay. With force and enthusiasm he proclaims that dental caries is not inevitable and is not the minor ailment that it passes for. We have not space to criticise Dr. Wallace's arguments, nor to specify the dietetic regulations which he regards as the first line of defence against dental caries. Anyone whose attention is attracted to the subject should obtain the collection of essays of which this book consists and read them for himself, and we will promise him a good reward for his toil. As to the gravity of dental decay there are many of us who know better than we like that it is no trival incident, and there is probably no one of a contemplative disposition who would urge the contrary.

The Consumptive Working Man. What Can Sanatoria Do For Him? By Noel Dean Bardswell, M.D., M.R.C.P., F.R.S. (Edin.), Medical Superintendent, King Edward VII. Sanatorium. (London: The Scientific Press, Limited, 28 and 29 Southampton Street, Strand, W.C.)

This is a most helpful and interesting book, alike for the practitioner and for the lay public, to whom the subject of consumption, its control and its cure, is of perennial interest. It is absolutely straightforward and unpretentious; one might truthfully describe it as a bare statement of facts, if it were not for the general assumption that bare statements of facts are dull, which Dr. Bardswell's book certainly is not. After a clear and simple description of the open-air wards of the Sheffield Royal Infirmary, and of the Mundesley Cottage Sanatorium, at one or other of which places the cases with which he deals were treated, he proceeds to give succinctly the details of each case—previous history, occupation, physical condition on entering and on leaving the hospital or sanatorium, length of treatment, and after record. The collection of these after-histories, running on, in some cases for five years, must have involved a very considerable amount of trouble, especially considering the class to which the patients belonged, but they form an exceedingly valuable part of the book and an answer to those who are indifferent to the establishing of sanatoria and find it convenient to say, "We can believe that while patients are under the practically ideal conditions of sanatorium life, their condition improves, but does the improvement continue after they return to their former environment?" In Dr. Bardswell's books we find out just how much a sanatorium can do for a man, and what must be done elsewhere. It is impossible to sum up in a brief review all the details which Dr. Bardswell has collected so carefully, but one may summarise them thus: Sanatorium treatment has always succeeded in greatly improving the patient's condition, and when the disease was taken early enough, has completely arrested it. In every case the patient's life and working powers have been prolonged, but to prevent a relapse it is necessary that the consumptive should be able to provide himself with a sufficiency of nourishing food, and should live in healthy—as much as possible open-air—conditions. Over-exertion, however, must be avoided; heavy farm work in one case and a long bicycle ride in another having brought on an attack of hæmoptysis. In several cases good work was done by societies or private philanthropists who maintained the families of patients during their treatment—thus relieving their minds of anxiety—and found them suitable light outdoor employment after their discharge. If it can be impressed on people that to sanatorium treatment must be added suitable after conditions, the value of these institutions will be much increased, and those interested in the common-sense treatment of consumption will find Dr. Bardswell's volume a convenient hand-book both for themselves and for any whom they may wish to instruct.

